# Supplementary material for: Aerobic Exercise Training with Brisk Walking Increases Intestinal Bacteroides in Healthy Elderly Women
Source: Nutrients. 2019 Apr 17;11(4):868. doi: 10.3390/nu11040868 (PMC6520866; doi:10.3390/nu11040868)
Supplement: Supplementary file 1 [file nutrients-11-00868-s001.pdf]

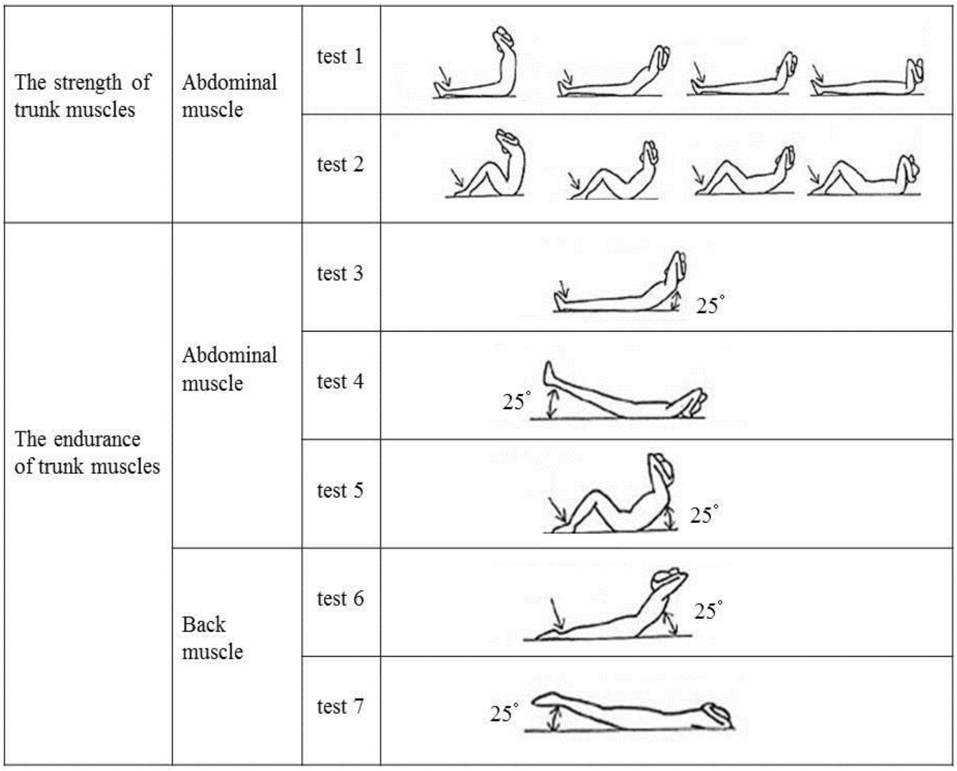

Figure 1. Modified Kraus–Weber test.

Strength-of-trunk-muscle test: the subject clasps his/her hands behind his/her head in the supine position with knees extended (test 1) or flexed (test 2). The subject is asked to roll up into a sitting position. Performance is rated based on a five-point scale (sitting up without support, 5 points; sitting up with support, 4 points; lumbar vertebrae not touching the floor, 3 points; scapula not touching the floor, 2 points; cervical vertebrae not touching the floor, 1 point). Endurance-of-trunk-muscle test: the subject in supine position with knees extended (test 3) or flexed (test 4). The examiner holds the subject's feet on the floor. The subject is asked to maintain posture with lifted head and shoulders off the floor for 60 s; the subject is further asked to lift his/her feet 25 cm above the floor, with knees extended, and maintain position for 60 s (test 5). Next, the subject in prone position with knees extended and hands clasped behind his/her head; the examiner holds the subject's feet, and the subject is asked to lift his/her chest and head and maintain position for 60 s (test 6). Finally, the subject is asked to lift without bending knees and maintain position for 60 s (test 7). Each component of the endurance of trunk muscle test was evaluated on a six-point scale (hold for ≥60 s, 6 points; for 50–59 s, 5 points; for 40–49 s, 4 points; for 30–39 s, 3 points; for 20–29 s, 2 points; for 10–19 s, 1 point; for ≤9 s, 0 points).

Reference: Koyama, Y.; Ishikawa, S.; Sukigara, S. Trunk Fitness of Female University Student evaluated by modified Kraus-Weber Test. Journal of Ibaraki Christian University. II, Social and natural sciences. 2007, 41, 211-220 (in Japanese).
